# Areca Nut and Oral Cancer: Evidence from Studies Conducted in Humans

**DOI:** 10.1177/00220345221092751

**Published:** 2022-04-22

**Authors:** S. Warnakulasuriya, T.H.H. Chen

**Affiliations:** 1 Faculty of Dentistry, Oral and Craniofacial Sciences, King’s College London, and the WHO Collaborating Centre for Oral Cancer, London, UK; 2 Institute of Epidemiology and Preventive Medicine, College of Public Health, National Taiwan University, Taipei

**Keywords:** betel quid, oral squamous cell carcinoma, oral potentially malignant disorders, arecoline, epidemiology, cessation

## Abstract

Areca nut chewing is one of the major risk factors for oral cancer, with large-magnitude risks reported in studies comparing betel quid chewers and never users, and it has been evaluated as a group 1 carcinogen by the International Agency for Research on Cancer. Data from a high-quality meta-analysis examining risk estimates are presented in summary form with additional information from more recent studies (pooled adjusted relative risk, 7.9; 95% CI, 7.1 to 8.7). The risk of oral cancer increases in a dose-response manner with the daily number of quids consumed and the number of years chewing. In the Indian subcontinent and in Taiwan, approximately half of oral cancers reported are attributed to betel quid chewing (population attributable fraction, 53.7% for residents in Taiwan and 49.5% for the Indian population), a disease burden that could be prevented. Oral leukoplakia and oral submucous fibrosis are 2 main oral potentially malignant disorders caused by areca nut chewing that can progress to oral cancer with continued use. Ex-chewers seem to demonstrate lower risks than current chewers, but the impact of areca nut cessation on oral cancer risk has not been scientifically evaluated on the basis of randomized controlled studies. These data strongly reconfirm that betel quid chewing, primarily areca nut use, should be taken into account in assessing the cancer risk of South Asian, East Asian populations and Pacific Islanders for the development of oral cancer.

## Introduction

The areca nut is the primary ingredient in betel quid (BQ), which is consumed by >600 million people in the world. BQ can be made up at home or purchased from venders as a ready-to-chew mix. A large variety of additives may be incorporated into the BQ according to one’s taste, but contents are usually betel leaf, areca nut, slaked lime, and sometimes spices. The most common method of adding areca nut to the quid mixture is to slice it into thin strips and roll it into a betel leaf with slaked lime (paste) or crushed seashells. The specific quid components vary among the communities and individuals who use it, due to its social and cultural patterns. Sun-dried tobacco is often added to the quid, but residents in Taiwan and Pacific Islanders generally consume BQ without tobacco. An association between BQ chewing and oral cancer was first identified in 1933 by a British surgeon based on a study of 100 cases of oral cancer in India (Orr 1933). Until the mid-1980s and due to the lack of data on pure areca nut chewers in the studies conducted in the Indian subcontinent, it was assumed that tobacco added to the quid was the carcinogenic agent in BQ (IARC Working Group 1985). The concept about the role of the areca nut (in the BQ) in the etiology for oral cancer initially emerged from Taiwan and South Africa. In Taiwan, with a high incidence of oral cancer, close to 50% of men consumed BQ and 80% of the preparations did not contain tobacco (Gupta and Warnakulasuriya 2002). From Natal, South Africa, 93% of women (83/87) diagnosed with oral squamous cell carcinoma habitually chewed areca nut (odds ratio [OR], 43.9; van Wyk et al. 1993). These observations along with emerging new evidence led the IARC in 2004 to reevaluate carcinogenicity of areca nut to humans.

## What Is Areca Nut?

Areca nut is the endosperm/seed of the areca fruit/drupe from the tropical palm tree *Areca catechu*, which grows in most parts of South Asia, much of the tropical regions of the Pacific Basin, and parts of East Africa. The fruit is ovoid or oblong with a pointed apex, and the outer surface (epicarp) is green when unripe and golden yellow when ripe. Areca nut is chewed by approximately 600 million people globally, most of whom live in low- to moderate-income countries in the Asia-Pacific region (Gupta and Warnakulasuriya 2002). The patterns of consumption vary. Areca nut is used at different stages of maturity: while the whole unripe fruit is consumed in Taiwan and the Pacific Islands, the ripe nut (separated from the pericarp of the drupe) is used in most parts of Asia. The nut can be used fresh, dried, and cured by baking, roasting, or sun drying. In mainland China, people consume the husk with the dried nut. A high prevalence of areca nut use, with or without tobacco, is also reported among immigrants from the Indian subcontinent living in the United States, United Kingdom, and Europe (Changrani et al. 2006; Petti and Warnakulasuriya 2018). Commercially manufactured freeze-dried products containing areca nut, such as Pan Parag, Pan Masala, or Guthka, have largely substituted the use of home-prepared fresh BQ. The easy availability of these commercially packaged products in colorful sachets has led to an increased use of areca nut.

Chewed BQ products remain in the oral cavity for prolonged periods. The most important oral mucosal lesions induced by the use of areca nut include oral cancer and oral potentially malignant disorders (OPMDs), such as oral leukoplakia, erythroplakia, oral lichenoid lesions, and oral submucous fibrosis. BQ chewers’ mucosa, although not included among OPMDs, is another oral mucosal lesion associated with BQ chewing.

BQ containing areca nut is the fourth-most frequently used addictive substance in the globe, following tobacco, alcohol, and caffeine. A dependency syndrome to areca nut chewing was first described by researchers at King’s College London (Winstock et al. 2000). Several subsequent epidemiologic surveys conducted in India and Pakistan and a multiethnic study in 6 countries in South Asia support the hypothesis of BQ dependence among BQ chewers (Benegal et al. 2008; Mirza et al. 2011; Lee et al. 2014).

## Chemical Constituents of Areca Nut

The major constituents of areca nut are carbohydrates, fats, proteins, crude fiber, polyphenols (flavonoids and tannins), alkaloids, and mineral matter. The constituents are region specific (Sari et al. 2020). Polyphenols constitute a large proportion of the dry weight and contribute to the astringent taste of the nut. Four alkaloids—arecoline, arecaidine, guvacine, and guvacoline—are important biologically. There is strong evidence from studies in human primary cells and various experimental systems that arecoline exhibits key characteristics of carcinogens (Tsai et al. 2008; Gupta et al. 2020). Areca nut–induced oral carcinogenesis is attributed to arecoline, reactive oxygen species, and nitrosamine. It has been shown that arecoline could induce DNA damages in human epithelial cells (Chang et al. 2001). Downregulation of p53, by arecoline, plays a critical role in the tumorigenesis of areca nut–associated malignancies (Tsai et al. 2008). Arecoline is genotoxic, inducing DNA strand breaks, micronucleus formation, chromosomal aberrations, and sister-chromatid exchanges in human primary and cultured cells (Gupta et al. 2020; IARC Monographs 2021). Based on mechanistic evidence, arecoline was classified as possibly carcinogenic to humans (group 2B) by the International Agency for Research on Cancer (IARC; IARC Monographs 2021). Lin et al. (2011) proposed that the mutagenic effects of arecoline may be due to one of its major metabolites: arecoline N-oxide.

The copper content of areca nut is much higher than that found in other nuts consumed by humans. Copper has been hypothesized to contribute to the fibrogenic activity of the nut (Trivedy et al. 1997). Experimental studies have implicated copper in areca nut in the causation of oral submucous fibrosis—an OPMD.

## Evidence for Carcinogenicity of Areca Nut in Humans

### Risk of Oral Cancer

Areca nut is an established cause of oral cancer and oral cancer–related deaths (Warnakulasuriya et al. 2002; IARC Working Group 2004, 2012). A meta-analysis of 26 observational studies published between 1933 and 2013 assessed the relationship between chewing BQ (with areca nut as the primary ingredient) and the risk of cancers of the oral cavity; it compiled evidence examined in the evaluation of the carcinogenicity of BQ in the IARC Monograph Volume 100E (IARC Working Group 2012) and was presented by Guha et al. (2014). All studies were conducted in the Indian subcontinent (*n* = 13) and Taiwan (*n* = 13). The meta–relative risk (mRR) for cancer of the oral cavity associated with chewing BQ generated summary relative risk estimates of 2.41 (95% CI, 1.82 to 3.19) for the Indian studies and 10.98 (95% CI, 4.86 to 24.84) for the Taiwan studies, with a moderate level of heterogeneity (*I*^2^ = 65%) for the Indian studies (Guha et al. 2014). Effect estimates for adding tobacco to the quid show greater magnitude of risk among Indian chewers (OR, 8.47; 95% CI, 6.49 to 11.05) than estimates for BQ alone. All studies assessing the joint effect of tobacco and BQ consumption have repeatedly shown a larger-than-multiplicative joint effect. When restricted to studies that adjusted for tobacco smoking, the mRR for BQ was 13.56 (95% CI, 6.57 to 28.00; 4 studies) in Taiwan and 2.94 (95% CI, 2.01 to 4.28; 8 studies) in the Indian subcontinent. Restricting the analysis to nonsmokers, the mRR for BQ was 20.21 (95% CI, 11.42 to 35.77; 2 studies) in Taiwan and 2.20 (95% CI, 1.32 to 3.66; 5 studies) in the Indian subcontinent. The mRR was much higher in women (mRR, 14.56; 95% CI, 7.63 to 27.76) than men in India. Effect estimates consistently showed a higher risk in the Taiwan population. This difference may be due to higher daily frequency of chewing in Taiwan but also to region-specific variations in the preparation of BQ, specifically in the unripe areca nut chewed and its preparation (Yang et al. 2001). A study in Taiwan demonstrated that retaining and subsequently swallowing BQ juice and including unripened whole areca fruit in the quid seemed to enhance the risks of contracting oral cancer by 11 times (Ko et al. 1995). Yang et al. (2021) proposed that disparity of study outcomes is to a large extent attributed to the differences of the components of BQ among geographic regions. Two other systematic reviews and meta-analysis have confirmed the carcinogenicity of betel quid without tobacco (Petti et al. 2013; Gupta and Johnson 2014).

Since 2014, after the last IARC evaluation and the publication of the meta-analyses referred to earlier, new evidence on the association of areca nut with oral cancer is available in the recently published literature based on 45 case-control studies and 8 cohort studies (Table 1). The pooled adjusted relative risk of these studies was 7.9 (95% CI, 7.1 to 8.7), which was slightly lower than the previous pooled estimate of 10.98 based on studies reported prior to 2014 (Guha et al. 2014). The lower pooled OR reported here could be due to better adjustments to covariates in the recent studies.

**Table 1.** Studies Reporting Risk of Oral Cancer in Betel Quid Chewers Without Tobacco (Since Last IARC Evaluation in 2014).

Reference	Country or Region	Study Design	Exposure	Odds Ratio (95% CI)
Chen et al. (2009)	Taiwan	Case-control study: 174 oral cancer cases and 347 controls	Chewer vs. nonchewer	20.1 (12.6 to 32.0)
Chung et al. (2009)	Taiwan	Case-control study: 160 oral cancer cases and 218 controls	Chewer vs. nonchewer	45.4 (21.1 to 97.5)
Kietthubthew et al. (2010)	Thailand	Case-control study: 107 oral cancer cases and 157 controls.	Betel quid chewer vs. nonchewer	1.9 (1.1 to 3.1)
Wang et al. (2010)	Taiwan	Case-control study: 294 oral cancer cases and 333 controls	Chewer vs. nonchewer	42.8 (26.9 to 67.9)
Chang et al. (2011)	Taiwan	Case-cohort study: 285 oral cancer cases and 13,321 subjects	Betel quid chewing vs. nonchewer	9.2 (2.8 to 30.7)
Chen et al. (2011a)	Taiwan	Case-control study: 216 oral cancer cases and 344 controls	Chewer vs. nonchewer	20.6 (13.3 to 31.9)^a^
Chen et al. (2011b)	Taiwan	Case-control study: 247 oral cancer cases and 338 controls	Chewer vs. nonchewer	17.3 (9.0 to 33.2)
Chung et al. (2011)	Taiwan	Case-control study: 415 oral cancer cases and 341 controls	Chewer vs. nonchewer	12.4 (8.8 to 17.4)
Lee et al. (2011)	South and East Asia	Cohort study: 1,522 subjects	Chewer vs. nonchewer	1.6 (1.3 to 2.0)^b^
Lin et al. (2011)	Taiwan	Cohort study: 10,657 subjects	Smoking, alcohol consumption, and betel quid chewing vs. none	34.8 (25.9 to 46.8)
Yuan et al. (2011)	Taiwan	Case-control study: 101 oral cancer cases and 104 controls	Chewer vs. nonchewer	16.0 (7.7 to 33.1)
Zavras et al. (2011)	Taiwan	Case-control study: 240 oral cancer cases and 347 controls	Chewer vs. nonchewer	19.9 (11.5 to 34.3)
Chen et al. (2012)	Taiwan	Case-control study: 444 oral cancer cases and 426 controls	Chewer vs. nonchewer	13.9 (10.0 to 19.3)
Chien et al. (2012)	Taiwan	Case-control study: 462 oral cancer cases and 519 controls	Chewer vs. nonchewer	15.2 (11.2 to 20.8)
Helen-Ng et al. (2012)	Malaysia	Case-control study: 153 oral cancer cases and 153 controls	Chewer vs. nonchewer	2.2 (1.3 to 3.8)
Lee et al. (2012)	South, Southeast, and East Asia	Case-control study: 810 oral cancer cases and 2,250 controls	Chewer vs. nonchewer	16.2 (12.1 to 21.7)
Lin et al. (2012a)	Taiwan	Case-control study: 462 oral cancer cases and 520 controls	Chewer vs. nonchewer	15.3 (11.2 to 20.9)
Lin et al. (2012b)	Taiwan	Case-control study: 195 oral cancer cases and 81 controls	Chewer vs. nonchewer	21.8 (10.6 to 44.8)
Liu et al. (2012)	Taiwan	Case-control study: 270 oral cancer cases and 350 controls	Chewer vs. nonchewer	21.0 (13.9 to 31.7)
Loyha et al. (2012)	Thailand	Case-control study: 104 oral cancer cases and 104 controls	Chewer vs. nonchewer	9.0 (3.8 to 21.2)
Madani et al. (2012)	India	Case-control study: 350 oral cancer cases and 350 controls	Chewer vs. nonchewer	6.6 (3.0 to 14.8)
Zavras et al. (2012)	Taiwan	Case-control study: 239 oral cancer cases and 336 controls	Chewer vs. nonchewer	20.1 (13.1 to 30.8)
Chien et al. (2013)	Taiwan	Case-control study: 470 oral cancer cases and 426 controls	Chewer vs. nonchewer	14.0 (10.1 to 19.3)
Tsai et al. (2014)	Taiwan	Case-control study: 788 oral cancer cases and 956 controls	Chewer vs. nonchewer	4.6 (3.7 to 5.8)
Wong et al. (2014)	Taiwan	Case-control study: 50 oral cancer cases and 50 controls	Chewer vs. nonchewer	1.3 (0.5 to 3.4)
Yang et al. (2014a)	Taiwan	Case-control study: 463 oral cancer cases and 623 controls	Chewer vs. nonchewer	8.1 (5.5 to 11.8)
Yang et al. (2014b)	Taiwan	Case-control study: 191 oral cancer cases and 100 controls	Chewer vs. nonchewer	14.7 (8.0 to 26.9)
Lee et al. (2015)	Taiwan	Case-control study: 507 oral cancer cases and 717 controls	Chewer vs. nonchewer	35.1 (25.6 to 48.3)
Chou et al. (2014)	Taiwan	Case-control study: 595 oral cancer cases and 561 controls	Chewer vs. nonchewer	16.5 (12.3 to 22.1)
Lin et al. (2015)	Taiwan	Case-control study: 618 oral cancer cases and 560 controls	Chewer vs. nonchewer	17.1 (12.8 to 23.0)
Su et al. (2015)	Taiwan	Case-control study: 747 oral cancer cases and 1,200 controls	Chewer vs. nonchewer	20.7 (16.4 to 26.2)
Chou et al. (2017a)	Taiwan	Case-control study: 876 oral cancer cases and 1,200 controls	Chewer vs. nonchewer	20.2 (16.1 to 25.2)
Chou et al. (2017b)	Taiwan	Case-control study: 955 oral cancer cases and 1,191 controls	Chewer vs. nonchewer	20.3 (16.3 to 25.4)
Chuang et al. (2017)	Taiwan	Cohort study: 2,334,299 subjects	Betel quid chewing without/with smoking vs. smoking only	2.8 (2.6 to 3.0)^a^
Chung et al. (2017a)	Taiwan	Case-control study: 447 oral cancer cases and 580 controls	Betel quid chewing vs. none.	26.7 (16.7 to 42.8)
Chung et al. (2017b)	Taiwan	Case-control study: 410 oral cancer cases and 282 controls	Chewer vs. nonchewer	28.5 (19.2 to 42.3)
Tsai et al. (2018), Shih et al. (2018)	Taiwan	Case-control study: 788 oral cancer cases and 956 controls	Chewer vs. nonchewer	5.9 (4.7 to 7.4)
Su et al. (2018)	Taiwan	Case-control study: 1,044 oral cancer cases and 1,200 controls	Chewer vs. nonchewer	16.5 (13.4 to 20.3)
Wu et al. (2018)	Taiwan	Cohort study: 310 subjects (malignant transformation for oral verrucous hyperplasia)	Betel quid chewing: (1) 10 to 20 and (2) >20 quids/d vs. <10	(1) 1.4 (0.8 to 2.5) and (2) 2.0 (1.3 to 4.0)^b^
Yang et al. (2018)	Taiwan	Case-control study: 935 oral cancer cases and 1,200 controls	Chewer vs. nonchewer	19.7 (15.8 to 24.6)
Huang et al. (2019)	Taiwan	Case-control study: 282 oral cancer cases and 324 controls	Chewer vs. nonchewer	37.0 (17.4 to 85.6)
Chen et al. (2019)	Taiwan	Case-control study: 242 oral cancer cases and 264 controls	Chewer vs. nonchewer	1.3 (0.8 to 2.2)
Chung et al. (2019)	Taiwan	Case-control study: 360 oral cancer cases and 486 controls	Chewer vs. nonchewer	26.8 (18.6 to 38.8)
Lin et al. (2019)	Taiwan	Case-control study: 741 oral cancer cases and 462 controls	Chewer vs. nonchewer	15.9 (11.9 to 21.3)
Su et al. (2019)	Taiwan	Cohort study: 5,743 subjects	Betel quid chewing without smoking vs. smoking only	1.2 (1.0 to 1.5)
Yen et al. (2019)	Taiwan	Cohort study: 235,234 subjects	Betel quid chewing with/without smoking vs. smoking only	2.2 (1.8 to 2.7)^b^
Shih et al. (2020), Wu et al. (2021)	Taiwan	Case-control study: 958 oral cancer cases and 958 controls	Chewer vs. nonchewer	3.7 (3.0 to 4.5)
Yeh et al. (2020)	Taiwan	Case-control study: 1,196 oral cancer cases and 1,200 controls	Chewer vs. nonchewer	13.7 (11.2 to 16.7)
Hu et al. (2020)	Mainland China	Case-control study: 304 cases and 304 controls	Chewer vs. nonchewer	5.4 (3.3 to 8.8)
Lin et al. (2020)	Mainland China	Cohort study: 915 subjects: (Metachronous multiple primary oral cancer)	Betel quid chewing without smoking	11.1 (9.23 to 13.3)
Chen et al. (2021)	Taiwan	Case-control study: 297 oral cancer cases and 193 controls	Chewer vs. nonchewer	13.3 (8.5 to 20.8)

For reference list, see Appendix.

IARC, International Agency for Research on Cancer.

a Values are presented as relative risk (95% CI).

b Values are presented as hazard ratio (95% CI).

Not only is areca nut carcinogenic to humans, but in a systematic review of 11 studies, areca nut users experienced a worse prognosis than nonchewers (Yang et al. 2021). Second primary cancers and local recurrences were also more common. Yang et al. (2021) explained that these aggressive outcomes in areca nut chewers could be due to oral submucous fibrosis of the underlying mucosa surrounding the malignancy (Yang et al. 2021).

### Attributable Risk: Areca Nut and Oral Cancer

It is important to examine how much of the oral cancer burden was due to BQ chewing. Based on the reported meta-analysis, the corresponding oral cancer population attributable burden was 53.7% for residents in Taiwan and 49.5% for the Indian population. However, the absolute number of cases annually in India (3,208 cases) is larger as compared with Taiwan (2,610 cases; Guha et al. 2014). Elimination of this lifestyle can reduce the risk among Indians substantially (by 50%) if all other factors remain the same. The evidence of attributable risk is an essential aspect for the design of effective oral cancer control policies by adopting appropriate interventions in Southeast Asia.

### Risk of OPMDs

The prevalence of OPMDs is much higher in Asia (10.54%; 95% CI, 4.60% to 18.55%) as compared with Europe (3.07%) or North America (0.11%) and significantly different to the overall global prevalence of OPMD (4.47%; Mello et al. 2018). This high prevalence in Asia is attributed to the abuse of areca nut by populations in the South Asia region (Lee et al. 2012). Table 2 lists all published studies on the effect of BQ chewing without tobacco on the risk of OPMD overall and the subtypes. A total of 7 cross-sectional studies, 12 case-control studies, and 2 cohort studies are shown in this table. For the outcome of OPMD, the range of ORs was between 1.36 (95% CI, 0.63 to 2.93) and 47.3 (95% CI, 26.8 to 83.6), yielding the pooled adjusted relative risk of 8.9 (95% CI, 7.9 to 10.0). As far as the subtypes of OPMD are concerned, a pooled OR of 2.8 (95% CI, 2.5 to 3.1) was estimated for leukoplakia with the range between 3.1 (95% CI, 2.5 to 3.7) and 48.6 (95% CI, 23.8 to 99.4). The corresponding pooled estimate was 25.7 (95% CI, 17.5 to 37.7) for oral submucosa fibrosis with the range between 8.7 (95% CI, 1.9 to 40.2) and 153.3 (95% CI, 34.4 to 683.4). All Taiwanese studies demonstrated a significant dose response with the risk for developing leukoplakia or submucous fibrosis, increased by the exposure level of chewing duration and quantity. Oral submucosa fibrosis shows specificity to areca nut use in a high proportion preceding oral cancer in areca nut chewers.

**Table 2.** Studies Reporting Risk of OPMD (Leukoplakia and Submucous Fibrosis) in Betel Quid Chewers Without Tobacco.

Reference	Country or Region	Study design	Exposure	Outcome	Odds Ratio (95% CI)
Shiu et al. (2000)	Taiwan	Case-control study: 100 leukoplakia and 100 control	Areca nut chewers vs. never	Leukoplakia	4.6 (1.3 to 16.9)
Pearson et al. (2001)	Bangladeshi living in the UK	Cross-sectional study: 185 subjects	Paan chewing vs. never	Leukoplakia	3.7 (0.9 to 15.1)
Lee et al. (2003)	Taiwan	Case-control study: 125 leukoplakia, 94 OSF, and 876 control	Areca nut chewers vs. never	(1) Leukoplakia	(1) 14.3 (8.4 to 24.6)
				(2) OSF	(2) 19.9 (12.5 to 31.8)
Jacob et al. (2004)	India	Case-control study: 927 leukoplakia, 170 OSF, 100 erythroplakia, and 47,773 control	Betel quid chewers vs. never	(1) Leukoplakia	(1) 4.0 (2.7 to 6.1)
				(2) OSF	(2) 47.2 (20.2 to 110.4)
				(3) Erythroplakia	(3) 12.5 (3.7 to 42.4)
Shiu et al. (2004)	Taiwan	Nested case-control study: 164 leukoplakia, 187 control	Areca nut chewers vs. never	Leukoplakia	17.7 (9.0 to 34.5)
Yang et al. (2005)	Taiwan	Case-control study: 62 OSF, 62 other oral mucosal lesion, and 62 control	Areca nut chewers vs. never	(1) OSF	(1) 8.7 (1.9 to 40.2)
				(2) Other oral mucosal lesion	(2) 8.4 (1.7 to 41.0)
Chung et al. (2005)	Taiwan	Cross-sectional study: 1,075 subjects	Areca nut chewers vs. never	OPMD	8.40 (5.13 to 13.75)
Yen et al. (2007)	Taiwan	Cohort study: 8,360 subjects	Areca nut chewers vs. never	(1) Leukoplakia	(1) 3.1 (2.5 to 3.7)
				(2) Erythroleukoplakia	(2) 12.5 (7.7 to 20.4)
Amarasinghe et al. (2010)	India	Case-control study: 17 OPMD and 411 control	Betel quid chewers vs. never	OPMD	5.5 (1.6 to 19.2)
Wang et al. (2010)	Taiwan	Case-control study: 53 OSF, 84 leukoplakia, and 333 control	Betel quid chewers vs. never	(1) OSF	(1) 35.8 (16.3 to 78.9)
				(2) Leukoplakia	(2) 48.6 (23.8 to 99.4)
Yang et al. (2010)	Taiwan	Cross-sectional study: 2,020 subjects	Areca nut chewers vs. never	(1) Leukoplakia for male	(1) 6.57 (3.51 to 12.28)^a^
				(2) Leukoplakia for female	(2) 15.63 (8.31 to 29.39)^a^
				(3) OSF for male	(3) 22.86 (7.28 to 71.73)^a^
				(4) OSF for female	(4) 13.03 (5.21 to 32.62)^a^
Yen et al. (2011)	Taiwan	Cohort study 79,940 subjects	Areca nut chewers vs. never	OPMD	19.7 (17.0 to 22.8)^a^
Lee et al. (2012)	Taiwan	Cross-sectional study: 1,548 subjects	Areca nut chewers vs. never	(1) OPMD	(1) 43.5 (11.6 to 162.7)
				(2) Submucous fibrosis (OSF)	(2) 24.4 (2.1 to —)
Lee et al. (2012)	Mainland China	Cross-sectional study: 2,356 subjects	Areca nut chewers vs. never	(1) OPMD	(1) 35.5 (13.7 to 91.8)
				(2) Submucous fibrosis (OSF)	(2) 153.3 (34.4 to 683.4)
				(3) Leukoplakia	(3) 25.5 (1.5 to 427.7)
Yang et al. (2014)	Taiwan	Case-control study: 30 OSF and 100 control	Areca nut chewers vs. never	OSF	27.7 (8.6 to 88.9)
Hsu et al. (2014)	Taiwan	Case-control study: 42 OPMD and 128 control	Areca nut chewers vs. never	OPMD	1.7 (0.7 to 3.9)
Juntanong et al. (2016)	Thailand	Cross-sectional study: 2,300 subjects	Areca nut chewers vs. never	OPMD	8.8 (3.2 to 24.5)
Zaw et al. (2016)	Myanmar	Cross-sectional study: 542 subjects	Areca nut chewers vs. never	OPMD	5.7 (1.4 to 22.9)
Chen et al. (2019)	Taiwan	Case-control study: 70 OPMD and 264 control	Areca nut chewers vs. never	OPMD	1.36 (0.63 to 2.93)
Huang et al. (2019)	Taiwan	Case-control study: 157 OPMD and 324 control	Areca nut chewers vs. never	OPMD	47.3 (26.8 to 83.6)
Chen et al. (2021)	Taiwan	Case-control study: 40 OPMD and 193 control	Areca nut chewers vs. never	OPMD	9.7 (4.1 to 23.2)

For reference list, see Appendix.

OPMD, oral potentially malignant disorder; OSF, oral submucosa fibrosis.

a Values are presented as hazard ratio (95% CI).

### Genetic Susceptibility

Only about 1% to 2% of BQ chewers develop OPMDs or oral cancer, suggesting the presence of some predisposition factor in these affected patients (IARC Working Group 2012). Among genetic studies, a specific association of BQ use and polymorphisms among patients with oral submucosa fibrosis relates to 6 collagen-related genes—collagen 1A1 and 1A2 (COL1A1 and COL1A2), collagenase 1 (COLase), transforming growth factor β1 (TGF-β1), lysyl oxidase (LYOXase), and cystatin C (CST3)—as reported by comparing patients with low and high exposure to BQ (Chiu et al. 2002). Other examples of genetic polymorphisms are in genes such as hypoxia-inducible factor 1α, V64I CCR2, CYP26B1, tumor necrosis factor α, CA9, VEGF-C, AURKA, FGFR4, and CD44 (Appendix Table). The assessment of gene-gene and gene-environment interactions requires large sample sizes to attain adequate statistical power, especially when the factors under study are very rare or very common or when the magnitude of the interaction is modest (Garcia-Closas et al. 2000).

### Impact of Cessation of Areca Nut Use

A limited number of behavioral and pharmacologic interventions to assist cessation of areca nut use have been reported recently (Moss et al. 2015; Hung, Lee, Chung, et al. 2020; Hung, Lee, Ko, et al. 2020). The total duration of these interventions was far too short to demonstrate any effectiveness on cancer incidence. There are no randomized controlled trials reported to assess the impact of BQ cessation on the risk of oral cancer. A national program to help areca nut users quit the BQ habit was reported from Taiwan. After significant increases in the past several decades, the age-standardized incidence rate of oral cancer has plateaued since 2009, 10 y since launching Areca Prevention Day (Yang et al. 2020).

A prospective cohort study conducted in India was designed to assess whether an educational intervention program led to the stoppage of chewing and resulted in the reduction of incident leukoplakia. The main finding after 10-y follow-up yielded a statistically significant reduction of the incidence of oral leukoplakia (Gupta et al. 1995).

By comparing the differences in the risk for OPMD or oral cancer between former and current chewers, the effectiveness of reducing and quitting exposure in reducing both outcomes can be inferred. Several studies found that the risk of OPMDs decreases with stopping areca nut use in comparison with continuing use. The prevalence of OPMDs in ex-chewers was lower than current chewers, but the differences were not statistically significant (Shiu et al. 2000). With reference to time since quitting, Wu et al. (2018) reported that the risk for oral cancer was substantially reduced for those quitters reporting cessation ≥20 y. The forthcoming IARC evaluation handbook on prevention of oral cancer demonstrates a statistically significant gradient relationship of reduced oral cancer risk with an increase in years of cessation and a more beneficial effect for those quitting ≥20 y when compared with current chewers and for those quitting at a younger age. The similar updated findings with even more remarkable effects were also observed for OPMD.

### Current Programs and Future Directions

Despite the currently available evidence that BQ and areca nut are known risk factors for oral pharyngeal and oesophageal cancers and their use is highly prevalent in the Asia-Pacific region, no global policy exists for the control of their use (Mehrtash et al. 2017). Unlike that for tobacco use, no systematic global or regional surveillance exists for BQ and areca nut use in their various forms. Taiwan is the only country in the Asia-Pacific region to introduce national policies to reduce areca nut use through educational and targeted cessation programs (Yang et al. 2020). Carcinogenicity of areca nut has not been widely communicated or acknowledged in most areca nut–consuming countries. Screening high-risk populations (i.e., areca nut users) should be explored to reduce the future incidence of oral cancer. Addressing the barriers to BQ and areca nut cessation is a multidisciplinary challenge (Athukorala et al. 2021) requiring the engagement of collaborators with diverse scientific expertise. Possible strategies to prevent BQ-associated cancer, including primary and secondary prevention, requires urgent attention. The WHO Global Oral Health Programme is planning to address the burden, challenges, and priority actions for renewing global commitment to improving oral health, including interventions to reduce the burden of oral cancer.

## Conclusion

The IARC has classified BQ (without added tobacco) and areca nut as group 1 carcinogens. The reported association between oral cancer and BQ chewing must therefore be seriously regarded in the design of effective oral cancer control policies in South Asia and the Pacific regions. A high percentage of BQ dependence has been acknowledged among BQ chewers, suggestive of the complex and challenging nature of BQ cessation. So far, formal interventional studies of high-quality randomized controlled trials focusing on primary prevention have not been undertaken to study the effectiveness of reducing or quitting BQ and areca nut use in reducing the incidence of OPMD and oral cancer.

## Author Contributions

S. Warnakulasuriya, contributed to conception, design and data acquisition, drafted and critically revised the manuscript; T.H.H. Chen, contributed to data acquisition and analysis, critically revised the manuscript. All authors gave final approval and agree to be accountable for all aspects of the work.

## Supplemental Material

sj-docx-1-jdr-10.1177_00220345221092751 – Supplemental material for Areca Nut and Oral Cancer: Evidence from Studies Conducted in Humans Click here for additional data file. Supplemental material, sj-docx-1-jdr-10.1177_00220345221092751 for Areca Nut and Oral Cancer: Evidence from Studies Conducted in Humans by S. Warnakulasuriya and T.H.H. Chen in Journal of Dental Research
