# Supplementary material for: Areca Nut and Oral Cancer: Evidence from Studies Conducted in Humans
Source: J Dent Res. 2022 Apr 22;101(10):1139–46. doi: 10.1177/00220345221092751 (PMC9397398; doi:10.1177/00220345221092751)
Supplement: sj-docx-1-jdr-10.1177_00220345221092751 – Supplemental material for Areca Nut and Oral Cancer: Evidence from Studies Conducted in Humans [file sj-docx-1-jdr-10.1177_00220345221092751.docx]

Supplementary Table 1: gene polymorphisms and increased susceptibility to oral cancer among BQ chewers

| Gene : polymorphisms or SNP | Description | Author (year) |
| --- | --- | --- |
| Hypoxia inducible factor-1alpha gene polymorphism | Among BQ and tobacco users compared to individuals with both C1772C and G1790G homozygotes, individuals with at least one of either C1772T or G1790A of HIF-1alpha gene had a risk of 2.17-folds (95% CI=1.0-4.75) to develop oral cancer. | Chen MK et al 2009 |
| V64I CCR2 gene polymorphism | 216 oral cancer patients (77.8% were areca nut chewers) after adjusting for other confounders, individuals with GA (aOR = 1.84; 95%CI = 1.10–3.20) or at least one A allele (aOR = 1.78; 95%CI = 1.05–3.02) had a higher risk for oral cancer, compared to GG genotype | Chen MK et al 2011 |
| CYP26B1 gene polymorphism | The independent risk of OSCC was observed among BQ chewers with CYP26B1 AA, compared with chewers with the CYP26B1 CC genotype (stratified aOR=2.88; 95% CI, 1.07-7.74). | Chen PH et al 2011 |
| SNPs in NOTCH1, BRCA1, COL9A1, and HSPA13 genes | Genetic risk scores for BQ chewers based on four tag SNPs were found in NOTCH1, BRCA1, COL9A1, and HSPA13 genes were significantly associated with OSCC occurrence (OR = 70.77; 95% CI, 8.70-575.73) after adjusting for other substance use and age | Chung CM et al., 2017a |
| Variants in NOTCH1 | The genetic variant rs139994842 in exon15 of NOTCH1 was significantly associated with an increased risk of OSCC (OR = 2.88 95% CI: 1.07-7.79) | Chung CM et al 2017b |
| tumour necrosis factor-α gene polymorphisms | G allele and G/G genotype at TNFA -308 were associated with a 1.95-fold (95%CI: 1.16-3.28, p=0.024) and 2.28-fold (95%CI: 1.30-4.00, p)=0.008) increased risk of cancer as compared to those with A allele or A/A+A/G genotypes, respectively. | Yang CM et al 2011 |
| Cathepsin B SNPs | three CTSB SNPs, combined with betel quid chewing [adjusted odds ratio (aOR) was 36.5, 21.7, and 43.9 for rs12338, rs13332, and rs8898, respectively] elevated the susceptibility to oral cancer. | Chen MK et al 2012 |
| CA9 gene polymorphisms | four CA9 SNPs rs2071676, rs3829078, rs1048638 and +376 Del combined with betel quid chewing and/or tobacco consumption elevate susceptibility to oral cancer | Chien MH et al 2012 |
| VEGF-C gene polymorphisms | In 470 male BQ chewers with oral cancer VEGF-C rs7664413 and rs2046463 polymorphisms were associated with oral-cancer susceptibility | Chien MH et al 2013 |
| Genetic polymorphisms of AURKA | In a total of 876 OSCC patients with the betel nut chewing habit, four SNPs of AURKA polymorphism carriers rs1047972, rs2273535, rs2064863, and rs6024836, , had a higher risk of oral cancer than AURKA wild-type (WT) carriers without the betel nut chewing habit | Chou CH et al (2017a)¦ |
| FGFR4 polymorphism | Among 955 patients with OSCC SNPs of FGFR4 (rs351855) GA genotype and a combination of the GA and AA genotypes exhibited a 1.431-fold (95% CI: 1.092-1.876) and 1.335-fold (95% CI: 1.033-1.725) higher risk of OSCC. | Chou CH et al (2017b) |
| CD44 gene polymorphisms | CD44 polymorphism carriers with the betel-nut chewing habit had a 10.30-37.63-fold greater risk of having oral cancer compared to CD44 wild-type (WT) carriers without the betel-nut chewing habit | Chou YE et al 2014 |
|  |  |  |

**References for supplementary Table 1**

Chen MK, Chiou HL, Su SC, Chung TT, Tseng HC, Tsai HT, Yang SF. The association between hypoxia inducible factor-1alpha gene polymorphisms and increased susceptibility to oral cancer. Oral Oncol. 2009 Dec;45(12):e222-6. doi: 10.1016/j.oraloncology.2009.07.015. Epub 2009 Aug 29. PMID: 19717330.

Chen MK, Yeh KT, Chiou HL, Lin CW, Chung TT, Yang SF. CCR2-64I gene polymorphism increase susceptibility to oral cancer. Oral Oncol. 2011 Jul;47(7):577-82. doi:10.1016/j.oraloncology.2011.04.008. Epub 2011 May 12. PMID: 21570337.

Chen PH, Lee KW, Chen CH, Shieh TY, Ho PS, Wang SJ, Lee CH, Yang SF, Chen MK, Chiang SL, Ko YC. CYP26B1 is a novel candidate gene for betel quid-related oral squamous cell carcinoma. Oral Oncol. 2011 Jul;47(7):594-600. doi: 10.1016/j.oraloncology.2011.04.024. Epub 2011 Jun 8. PMID: 21641851.

Chung CM, Lee CH, Chen MK, Lee KW, Lan CE, Kwan AL, Tsai MH, Ko YC. Combined Genetic Biomarkers and Betel Quid Chewing for Identifying High-Risk Group for Oral Cancer Occurrence. Cancer Prev Res (Phila). 2017a Jun;10(6):355-362. doi: 10.1158/1940-6207.CAPR-16-0259. Epub 2017 Apr 11. PMID: 28400480.

Chung CM, Lee CH, Chen MK, Tsai MH, Ko YC. Interaction Between Rare Variants in NOTCH1 and Betel Quid Chewing in Oral Squamous Cell Carcinoma. Genet Test Mol Biomarkers. 2017 b Oct;21(10):608-612. doi: 10.1089/gtmb.2017.0013. Epub 2017 Sep 14. PMID: 28910158.

Yang CM, Hou YY, Chiu YT, Chen HC, Chu ST, Chi CC, Hsiao M, Lee CY, Hsieh CJ, Lin YC, Hsieh YD, Ger LP. Interaction between tumour necrosis factor-α gene polymorphisms and substance use on risk of betel quid-related oral and pharyngeal squamous cell carcinoma in Taiwan. Arch Oral Biol. 2011 Oct;56(10):1162-9. doi: 10.1016/j.archoralbio.2011.03.009. Epub 2011 Apr 16. PMID: 21497332.

Chen MK, Su SC, Lin CW, Tsai CM, Yang SF, Weng CJ. Cathepsin B SNPs elevate the pathological development of oral cancer and raise the susceptibility to carcinogen-mediated oral cancer. Hum Genet. 2012 Dec;131(12):1861-8. doi: 10.1007/s00439-012-1211-1. Epub 2012 Aug 1. PMID: 22851129.

Chien MH, Yang JS, Chu YH, Lin CH, Wei LH, Yang SF, Lin CW. Impacts of CA9 gene polymorphisms and environmental factors on oral-cancer susceptibility and clinicopathologic characteristics in Taiwan. PLoS One. 2012;7(12):e51051. doi: 10.1371/journal.pone.0051051. Epub 2012 Dec 4. PMID: 23226559; PMCID: PMC3514272.

Chien MH, Liu YF, Hsin CH, Lin CH, Shih CH, Yang SF, Cheng CW, Lin CW. Impact of VEGF-C gene polymorphisms and environmental factors on oral cancer susceptibility in Taiwan. PLoS One. 2013 Apr 4;8(4):e60283. doi: 10.1371/journal.pone.0060283. PMID: 23593187; PMCID: PMC3617207.

Chou CH, Chou YE, Chuang CY, Yang SF, Lin CW. Combined effect of genetic polymorphisms of AURKA and environmental factors on oral cancer development in Taiwan. PLoS One. 2017a Feb 2;12(2):e0171583. doi: 10.1371/journal.pone.0171583. PMID: 28152093; PMCID: PMC5289639.

Chou CH, Hsieh MJ, Chuang CY, Lin JT, Yeh CM, Tseng PY, Yang SF, Chen MK, Lin CW. Functional FGFR4 Gly388Arg polymorphism contributes to oral squamous cell carcinoma susceptibility. Oncotarget. 2017b Oct 23;8(56):96225-96238. doi: 10.18632/oncotarget.21958. PMID: 29221201; PMCID: PMC5707095.

Chou YE, Hsieh MJ, Hsin CH, Chiang WL, Lai YC, Lee YH, Huang SC, Yang SF, Lin CW. CD44 gene polymorphisms and environmental factors on oral cancer susceptibility in Taiwan. PLoS One. 2014 Apr 3;9(4):e93692. doi: 10.1371/journal.pone.0093692. PMID: 24699672; PMCID: PMC3974805.

**References cited for Table 1, Studies reporting risk of oral cancer in betel quid chewers without tobacco.**

Chang IH, Jiang RS, Wong YK, Wu SH, Chen FJ, Liu SA (2011). Visual screening of oral cavity cancer in a male population: experience from a medical center. J Chin Med Assoc. 74(12):561–6. https://doi.org/10.1016/j.jcma.2011.09.014 PMID:22196472

Chen MK, Chiou HL, Su SC, Chung TT, Tseng HC, Tsai HT, et al. (2009). The association between hypoxia inducible factor-1alpha gene polymorphisms and increased susceptibility to oral cancer. Oral Oncol. 45(12):e222–6. https://doi.org/10.1016/j.oraloncology.2009.07.015 PMID:19717330

Chen MK, Yeh KT, Chiou HL, Lin CW, Chung TT, Yang SF (2011a). CCR2–64I gene polymorphism increase susceptibility to oral cancer. Oral Oncol. 47(7):577–82. https://doi.org/10.1016/j.oraloncology.2011.04.008 PMID:21570337

Chen PH, Lee KW, Chen CH, Shieh TY, Ho PS, Wang SJ, et al. (2011b). CYP26B1 is a novel candidate gene for betel quid-related oral squamous cell carcinoma. Oral Oncol. 47(7):594–600. https://doi.org/10.1016/j.oraloncology.2011.04.024 PMID:21641851

Chen PH, Chuang LY, Wu KC, Wang YH, Shieh TY, Sheu JJ, et al. (2019). Application of simulation-based CYP26 SNP-environment barcodes for evaluating the occurrence of oral malignant disorders by odds ratio-based binary particle swarm optimization: A case-control study in the Taiwanese population. PLoS One. 14(8):e0220719. https://doi.org/10.1371/journal.pone.0220719 PMID:31465460

Chen MK, Su SC, Lin CW, Tsai CM, Yang SF, Weng CJ (2012). Cathepsin B SNPs elevate the pathological development of oral cancer and raise the susceptibility to carcinogen-mediated oral cancer. Hum Genet. 131(12):1861–8. https://doi.org/10.1007/s00439-012-1211-1 PMID:22851129

Chen PH, Wang YY, Lan TH, Chan LP, Yuan SS (2021). Genetic and Proteinic Linkage of MAO and COMT with Oral Potentially Malignant Disorders and Cancers of the Oral Cavity and Pharynx. Cancers (Basel). 13(13):3268. https://doi.org/10.3390/cancers13133268 PMID:34209963

Chien MH, Yang JS, Chu YH, Lin CH, Wei LH, Yang SF, et al. (2012). Impacts of CA9 gene polymorphisms and environmental factors on oral-cancer susceptibility and clinicopathologic characteristics in Taiwan. PLoS One. 7(12):e51051. https://doi.org/10.1371/journal.pone.0051051 PMID:23226559

Chien MH, Liu YF, Hsin CH, Lin CH, Shih CH, Yang SF, et al. (2013). Impact of VEGF-C gene polymorphisms and environmental factors on oral cancer susceptibility in Taiwan. PLoS One. 8(4):e60283. https://doi.org/10.1371/journal.pone.0060283 PMID:23593187

Chou CH, Chou YE, Chuang CY, Yang SF, Lin CW (2017a). Combined effect of genetic polymorphisms of AURKA and environmental factors on oral cancer development in Taiwan. PLoS One. 12(2):e0171583. https://doi.org/10.1371/journal.pone.0171583 PMID:28152093

Chou CH, Hsieh MJ, Chuang CY, Lin JT, Yeh CM, Tseng PY, et al. (2017b). Functional FGFR4 Gly388Arg polymorphism contributes to oral squamous cell carcinoma susceptibility. Oncotarget. 8(56):96225–38. https://doi.org/10.18632/oncotarget.21958 PMID:29221201

Chou YE, Hsieh MJ, Hsin CH, Chiang WL, Lai YC, Lee YH, et al. (2014). CD44 gene polymorphisms and environmental factors on oral cancer susceptibility in Taiwan. PLoS One. 9(4):e93692. https://doi.org/10.1371/journal.pone.0093692 PMID:24699672

Chuang SL, Su WW, Chen SL, Yen AM, Wang CP, Fann JC, et al. (2017). Population-based screening program for reducing oral cancer mortality in 2,334,299 Taiwanese cigarette smokers and/or betel quid chewers. Cancer. 123(9):1597–609. https://doi.org/10.1002/cncr.30517 PMID:28055109

Chung CM, Lee CH, Chen MK, Lee KW, Lan CE, Kwan AL, et al. (2017a). Combined Genetic Biomarkers and Betel Quid Chewing for Identifying High-Risk Group for Oral Cancer Occurrence. Cancer Prev Res (Phila). 10(6):355–62. https://doi.org/10.1158/1940-6207.CAPR-16-0259 PMID:28400480

Chung CM, Lee CH, Chen MK, Tsai MH, Ko YC (2017b). Interaction Between Rare Variants in NOTCH1 and Betel Quid Chewing in Oral Squamous Cell Carcinoma. Genet Test Mol Biomarkers. 21(10):608–12. https://doi.org/10.1089/gtmb.2017.0013 PMID:28910158

Chung YT, Hsieh LL, Chen IH, Liao CT, Liou SH, Chi CW, et al. (2009). Sulfotransferase 1A1 haplotypes associated with oral squamous cell carcinoma susceptibility in male Taiwanese. Carcinogenesis. 30(2):286–94. https://doi.org/10.1093/carcin/bgn283 PMID:19126640

Chung TT, Pan MS, Kuo CL, Wong RH, Lin CW, Chen MK, et al. (2011). Impact of RECK gene polymorphisms and environmental factors on oral cancer susceptibility and clinicopathologic characteristics in Taiwan. Carcinogenesis. 32(7):1063–8. https://doi.org/10.1093/carcin/bgr083 PMID:21565829

Chung CM, Hung CC, Lee CH, Lee CP, Lee KW, Chen MK, et al. (2019). Variants in FAT1 and COL9A1 genes in male population with or without substance use to assess the risk factors for oral malignancy. PLoS One. 14(1):e0210901. https://doi.org/10.1371/journal.pone.0210901 PMID:30657779

Helen-Ng LC, Razak IA, Ghani WM, Marhazlinda J, Norain AT, Raja Jallaludin RL, et al. (2012). Dietary pattern and oral cancer risk–a factor analysis study. Community Dent Oral Epidemiol. 40(6):560–6. https://doi.org/10.1111/j.1600-0528.2012.00704.x PMID:22679921

Hu Y, Zhong R, Li H, Zou Y (2020). Effects of Betel Quid, Smoking and Alcohol on Oral Cancer Risk: A Case-Control Study in Hunan Province, China. Subst Use Misuse. 55(9):1501–8. https://doi.org/10.1080/10826084.2020.1750031 PMID:32569534

Huang HI, Chen CH, Wang SH, Wang LH, Lin YC (2019). Effects of APE1 Asp148Glu polymorphisms on OPMD malignant transformation, and on susceptibility to and overall survival of oral cancer in Taiwan. Head Neck. 41(6):1557–64. https://doi.org/10.1002/hed.25576 PMID:30652382

Kietthubthew S, Wickliffe J, Sriplung H, Ishida T, Chonmaitree T, Au WW (2010). Association of polymorphisms in proinflammatory cytokine genes with the development of oral cancer in Southern Thailand. Int J Hyg Environ Health. 213(2):146–52. https://doi.org/10.1016/j.ijheh.2010.01.002 PMID:20133197

Lee CH, Ko AMS, Warnakulasuriya S, Ling TY, Sunarjo, Rajapakse PS, et al. (2012). Population burden of betel quid abuse and its relation to oral premalignant disorders in South, Southeast, and East Asia: an Asian Betel-quid Consortium Study. Am J Public Health. 102(3):e17–24. https://doi.org/10.2105/AJPH.2011.300521 PMID:22390524

Lee CP, Chiang SL, Lee CH, Tsai YS, Wang ZH, Hua CH, et al. (2015). AURKA Phe31Ile polymorphism interacted with use of alcohol, betel quid, and cigarettes at multiplicative risk of oral cancer occurrence. Clin Oral Investig. 19(8):1825–32. https://doi.org/10.1007/s00784-015-1432-5 PMID:25697104

Lin FY, Lin CW, Yang SF, Lee WJ, Lin YW, Lee LM, et al. (2015). Interactions between environmental factors and melatonin receptor type 1A polymorphism in relation to oral cancer susceptibility and clinicopathologic development. PLoS One. 10(3):e0121677. https://doi.org/10.1371/journal.pone.0121677 PMID:25806809

Lin CW, Hsieh YS, Hsin CH, Su CW, Lin CH, Wei LH, et al. (2012a). Effects of NFKB1 and NFKBIA gene polymorphisms on susceptibility to environmental factors and the clinicopathologic development of oral cancer. PLoS One. 7(4):e35078. https://doi.org/10.1371/journal.pone.0035078 PMID:22509384

Lin CW, Tseng SW, Yang SF, Ko CP, Lin CH, Wei LH, et al. (2012b). Role of lipocalin 2 and its complex with matrix metalloproteinase-9 in oral cancer. Oral Dis. 18(8):734–40. https://doi.org/10.1111/j.1601-0825.2012.01938.x PMID:22533572

Lee CH, Ko AMS, Warnakulasuriya S, Yin BL, Zain RB, Ibrahim SO et al. (2011). Intercountry prevalences and practices of betel‐quid use in south, southeast and eastern Asia regions and associated oral preneoplastic disorders: an international collaborative study by Asian betel‐quid consortium of south and east Asia. International Journal of Cancer, 129(7), 1741-1751. https://10.1002/ijc.25809 PMID: 21128235

Lin SH, Chen MK, Chang JH, Velmurugan BK, Annamanedi M, Su SC, et al. (2019). Impact of Polymorphisms in Casein Kinase 1 Epsilon and Environmental Factors in Oral Cancer Susceptibility. J Cancer. 10(21):5065–9. https://doi.org/10.7150/jca.34592 PMID:31602258

Liu CM, Yeh CJ, Yu CC, Chou MY, Lin CH, Wei LH, et al. (2012). Impact of interleukin-8 gene polymorphisms and environmental factors on oral cancer susceptibility in Taiwan. Oral Dis. 18(3):307–14. https://doi.org/10.1111/j.1601-0825.2011.01882.x PMID:22151543

Lin X, Wu X, Gomaa A, Chen J, Wu L, Xie X et al (2020) . Analysis of risk factors for multiple primary oral squamous cell carcinoma: a cohort study. Clin Oral Investing. 24(9): 3147-3155. https:// doi: 10.1007/s00784-019-03189-0 PMID: 31903501

Lin WJ, Jiang RS, Wu SH, Chen FJ, Liu SA (2011). Smoking, alcohol, and betel quid and oral cancer: a prospective cohort study. J Oncol.525976. https://doi.org/10.1155/2011/525976 PMID:21547265

Loyha K, Vatanasapt P, Promthet S, Parkin DM (2012). Risk factors for oral cancer in northeast Thailand. Asian Pac J Cancer Prev. 13(10):5087–90. https://doi.org/10.7314/APJCP.2012.13.10.5087 PMID:23244115

Madani AH, Dikshit M, Bhaduri D (2012). Risk for oral cancer associated to smoking, smokeless and oral dip products. Indian J Public Health. 56(1):57–60. https://doi.org/10.4103/0019-557X.96977 PMID:22684175

Shih LC, Li CH, Sun KT, Chen LY, Hsu CL, Hung YW, et al. (2018). Association of Matrix Metalloproteinase-7 Genotypes to the Risk of Oral Cancer in Taiwan. Anticancer Res. 38(4):2087–92. 10.21873/anticanres.12448 PMID:29599326

Su CW, Huang YW, Chen MK, Su SC, Yang SF, Lin CW (2015). Polymorphisms and Plasma Levels of Tissue Inhibitor of Metalloproteinase-3: Impact on Genetic Susceptibility and Clinical Outcome of Oral Cancer. Medicine (Baltimore). 94(46):e2092. https://doi.org/10.1097/MD.0000000000002092 PMID:26579821

Su CW, Chien MH, Lin CW, Chen MK, Chow JM, Chuang CY, et al. (2018). Associations of genetic variations of the endothelial nitric oxide synthase gene and environmental carcinogens with oral cancer susceptibility and development. Nitric Oxide. 79:1–7. https://doi.org/10.1016/j.niox.2018.06.005 PMID:29932969

Su WW, Su CW, Chang DC, Chuang SL, Chen SL, Hsu CY, et al. (2019). Impact of varying anatomic sites on advanced stage and survival of oral cancer: 9-year prospective cohort of 27 717 cases. Head Neck. 41(5):1475–83. https://doi.org/10.1002/hed.25579 PMID:30652378

Tsai CW, Chang WS, Liu JC, Tsai MH, Lin CC, Bau DT (2014). Contribution of DNA double-strand break repair gene XRCC3 genotypes to oral cancer susceptibility in Taiwan. Anticancer Res. 34(6):2951–6. PMID:24922659</jrn>

Tsai CW, Hsu HM, Wang YC, Chang WS, Shih LC, Sun KT, et al. (2018). Contribution of MMP2 Promoter Genotypes to Oral Cancer Susceptibility, Recurrence and Metastasis in Taiwan. Anticancer Res. 38(12):6821–6. https://doi.org/10.21873/anticanres.13055 PMID:30504396

Wang LH, Ting SC, Chen CH, Tsai CC, Lung O, Liu TC, et al. (2010). Polymorphisms in the apoptosis-associated genes FAS and FASL and risk of oral cancer and malignant potential of oral premalignant lesions in a Taiwanese population. J Oral Pathol Med. 39(2):155–61. https://doi.org/10.1111/j.1600-0714.2009.00873.x PMID:20359312

Wong GR, Ha KO, Himratul-Aznita WH, Yang YH, Wan Mustafa WM, Yuen KM, et al. (2014). Seropositivity of HPV 16 E6 and E7 and the risk of oral cancer. Oral Dis. 20(8):762–7. https://doi.org/10.1111/odi.12218 PMID:24320099

Wu MH, Luo JD, Wang WC, Chang TH, Hwang WL, Lee KH, et al. (2018). Risk analysis of malignant potential of oral verrucous hyperplasia: A follow-up study of 269 patients and copy number variation analysis. Head Neck. 40(5):1046–56. https://doi.org/10.1002/hed.25076 PMID:29377391

Yang CM, Chen HC, Hou YY, Lee MC, Liou HH, Huang SJ, et al. (2014a). A high IL-4 production diplotype is associated with an increased risk but better prognosis of oral and pharyngeal carcinomas. Arch Oral Biol. 59(1):35–46. https://doi.org/10.1016/j.archoralbio.2013.09.010 PMID:24169152

Yang JS, Chen MK, Yang SF, Chang YC, Su SC, Chiou HL, et al. (2014b). Increased expression of carbonic anhydrase IX in oral submucous fibrosis and oral squamous cell carcinoma. Clin Chem Lab Med. 52(9):1367–77. https://doi.org/10.1515/cclm-2014-0129 PMID:24695043

Yang WH, Wang SJ, Chang YS, Su CM, Yang SF, Tang CH (2018). Association of Resistin Gene Polymorphisms with Oral Squamous Cell Carcinoma Progression and Development. BioMed Res Int. 2018:9531315. https://doi.org/10.1155/2018/9531315 PMID:30406149

Yen AM, Wang ST, Feng SW, Lin CT, Chen SL (2019). The association between fecal hemoglobin concentration and oral potentially malignant disorders. Oral Dis. 25(1):108–16. https://doi.org/10.1111/odi.12978 PMID:30216606

Yuan TH, Lian IeB, Tsai KY, Chang TK, Chiang CT, Su CC, et al. (2011). Possible association between nickel and chromium and oral cancer: a case-control study in central Taiwan. Sci Total Environ. 409(6):1046–52. https://doi.org/10.1016/j.scitotenv.2010.11.038 PMID:21195455

Zavras AI, Yoon AJ, Chen MK, Lin CW, Yang SF (2011). Metallothionein-1 genotypes in the risk of oral squamous cell carcinoma. Ann Surg Oncol. 18(5):1478–83. https://doi.org/10.1245/s10434-010-1431-3 PMID:21128001

Zavras AI, Yoon AJ, Chen MK, Lin CW, Yang SF (2012). Association between polymorphisms of DNA repair gene ERCC5 and oral squamous cell carcinoma. Oral Surg Oral Med Oral Pathol Oral Radiol. 114(5):624–9. https://doi.org/10.1016/j.oooo.2012.05.013 PMID:22981091

**References cited for Table 2, Studies reporting risk of OPMD (leukoplakia & submucous fibrosis) in betel quid chewers without tobacco**

Amarasinghe HK, Usgodaarachchi US, Johnson NW, Lalloo R, Warnakulasuriya S (2010). Betel-quid chewing with or without tobacco is a major risk factor for oral potentially malignant disorders in Sri Lanka: a case-control study. Oral Oncol. 46(4):297–301. https://doi.org/10.1016/j.oraloncology.2010.01.017 PMID:20189448

Chen PH, Chuang LY, Wu KC, Wang YH, Shieh TY, Sheu JJ, et al. (2019). Application of simulation-based CYP26 SNP-environment barcodes for evaluating the occurrence of oral malignant disorders by odds ratio-based binary particle swarm optimization: A case-control study in the Taiwanese population. PLoS One. 14(8):e0220719. https://doi.org/10.1371/journal.pone.0220719 PMID:31465460

Chen PH, Wang YY, Lan TH, Chan LP, Yuan SS (2021). Genetic and Proteinic Linkage of MAO and COMT with Oral Potentially Malignant Disorders and Cancers of the Oral Cavity and Pharynx. Cancers (Basel). 13(13):3268. https://doi.org/10.3390/cancers13133268 PMID:34209963

Chung CH, Yang YH, Wang TY, Shieh TY, Warnakulasuriya S (2005). Oral precancerous disorders associated with areca quid chewing, smoking, and alcohol drinking in southern Taiwan. J Oral Pathol Med. 34(8):460–6. https://doi.org/10.1111/j.1600-0714.2005.00332.x PMID:16091112

Hsu HJ, Yang YH, Shieh TY, Chen CH, Kao YH, Yang CF, et al. (2014). Role of cytokine gene (interferon-γ, transforming growth factor-β1, tumor necrosis factor-α, interleukin-6, and interleukin-10) polymorphisms in the risk of oral precancerous lesions in Taiwanese. Kaohsiung J Med Sci. 30(11):551–8. https://doi.org/10.1016/j.kjms.2014.09.003 PMID:25458044

Huang HI, Chen CH, Wang SH, Wang LH, Lin YC (2019). Effects of APE1 Asp148Glu polymorphisms on OPMD malignant transformation, and on susceptibility to and overall survival of oral cancer in Taiwan. Head Neck. 41(6):1557–64. https://doi.org/10.1002/hed.25576 PMID:30652382

Jacob BJ, Straif K, Thomas G, Ramadas K, Mathew B, Zhang ZF, et al. (2004). Betel quid without tobacco as a risk factor for oral precancers. Oral Oncol. 40(7):697–704. https://doi.org/10.1016/j.oraloncology.2004.01.005 PMID:15172639

Juntanong N, Siewchaisakul P, Bradshaw P, Vatanasapt P, Chen SL, Yen AM, et al. (2016). Prevalence and Factors Associated with Oral Pre-Malignant Lesions in Northeast Thailand. Asian Pac J Cancer Prev. 17(8):4175–9. PMID:27644680

Lee CH, Ko YC, Huang HL, Chao YY, Tsai CC, Shieh TY, et al. (2003). The precancer risk of betel quid chewing, tobacco use and alcohol consumption in oral leukoplakia and oral submucous fibrosis in southern Taiwan. Br J Cancer. 88(3):366–72. https://doi.org/10.1038/sj.bjc.6600727 PMID:12569378

Lee CH, Ko AMS, Warnakulasuriya S, Ling TY, Sunarjo, Rajapakse PS, et al. (2012a). Population burden of betel quid abuse and its relation to oral premalignant disorders in South, Southeast, and East Asia: an Asian Betel-quid Consortium Study. Am J Public Health. 102(3):e17–24. https://doi.org/10.2105/AJPH.2011.300521 PMID:22390524

Lee CH, Ko AMS, Warnakulasuriya S, Ling TY, Sunarjo, Rajapakse PS, et al. (2012b). Population burden of betel quid abuse and its relation to oral premalignant disorders in South, Southeast, and East Asia: an Asian Betel-quid Consortium Study. Am J Public Health. 102(3):e17–24. https://doi.org/10.2105/AJPH.2011.300521 PMID:22390524

Pearson N, Croucher R, Marcenes W, O’Farrell M (2001). Prevalence of oral lesions among a sample of Bangladeshi medical users aged 40 years and over living in Tower Hamlets, UK. Int Dent J. 51(1):30–4. https://doi.org/10.1002/j.1875-595X.2001.tb00814.x PMID:11326446

Shiu MN, Chen TH, Chang SH, Hahn LJ (2000). Risk factors for leukoplakia and malignant transformation to oral carcinoma: a leukoplakia cohort in Taiwan. Br J Cancer. 82(11):1871–4. https://doi.org/10.1054/bjoc.2000.1208 PMID:10839305

Shiu MN, Chen TH (2004). Impact of betel quid, tobacco and alcohol on three-stage disease natural history of oral leukoplakia and cancer: implication for prevention of oral cancer. Eur J Cancer Prev. 13(1):39–45. https://doi.org/10.1097/00008469-200402000-00007 PMID:15075787

Wang LH, Ting SC, Chen CH, Tsai CC, Lung O, Liu TC, et al. (2010). Polymorphisms in the apoptosis-associated genes FAS and FASL and risk of oral cancer and malignant potential of oral premalignant lesions in a Taiwanese population. J Oral Pathol Med. 39(2):155–61. https://doi.org/10.1111/j.1600-0714.2009.00873.x PMID:20359312

Yang YH, Lien YC, Ho PS, Chen CH, Chang JS, Cheng TC, et al. (2005). The effects of chewing areca/betel quid with and without cigarette smoking on oral submucous fibrosis and oral mucosal lesions. Oral Dis. 11(2):88–94. https://doi.org/10.1111/j.1601-0825.2004.01061.x PMID:15752081

Yang YH, Ho PS, Lu HM, Huang IY, Chen CH (2010). Comparing dose-response measurements of oral habits on oral leukoplakia and oral submucous fibrosis from a community screening program. J Oral Pathol Med. 39(4):306–12. https://doi.org/10.1111/j.1600-0714.2009.00820.x PMID:20149061

Yang CM, Chen HC, Hou YY, Lee MC, Liou HH, Huang SJ, et al. (2014). A high IL-4 production diplotype is associated with an increased risk but better prognosis of oral and pharyngeal carcinomas. Arch Oral Biol. 59(1):35–46. https://doi.org/10.1016/j.archoralbio.2013.09.010 PMID:24169152

Yen AM, Chen SC, Chen TH (2007). Dose-response relationships of oral habits associated with the risk of oral pre-malignant lesions among men who chew betel quid. Oral Oncol. 43(7):634–8. https://doi.org/10.1016/j.oraloncology.2006.05.001 PMID:17466570

Yen AM, Chen SL, Chiu SY, Chen HH (2011). Association between metabolic syndrome and oral pre-malignancy: a community- and population-based study (KCIS No. 28). Oral Oncol. 47(7):625–30. https://doi.org/10.1016/j.oraloncology.2011.04.011 PMID:21592847

Zaw KK, Ohnmar M, Hlaing MM, Oo YT, Win SS, Htike MM, et al. (2016). Betel Quid and Oral Potentially Malignant Disorders in a Periurban Township in Myanmar. PLoS One. 11(9):e0162081. https://doi.org/10.1371/journal.pone.0162081 PMID:27611195
